# Role of Low Ankle–Brachial Index in Cardiovascular and Mortality Risk Compared with Major Risk Conditions

**DOI:** 10.3390/jcm8060870

**Published:** 2019-06-18

**Authors:** Lia Alves-Cabratosa, Maria Garcia-Gil, Marc Comas-Cufí, Jordi Blanch, Anna Ponjoan, Ruth Martí-Lluch, Marc Elosua-Bayes, Dídac Parramon, Lourdes Camós, Rafel Ramos

**Affiliations:** 1 Vascular Health Research Group of Girona (ISV-Girona), Fundació Institut Universitari d’Investigació en Atenció Primària Jordi Gol (IDIAP Jordi Gol), 17002 Girona, Spain; lalves@idiapjgol.info (L.A.-C.); mgarcia@idiapjgol.info (M.G.-G.); mcomas@idiapjgol.info (M.C.-C.); jblanch@idiapjgol.info (J.B.); aponjoan@idiapjgol.info (A.P.); rmarti.girona.ics@gencat.cat (R.M.-L.); melosua@idiapjgol.info (M.E.-B.); dparramont.girona.ics@gencat.cat (D.P.); lourdes5d8@gmail.com (L.C.); 2 Department of Medical Sciences, School of Medicine, University of Girona, 17003 Girona, Spain; 3 Institut d’Investigació Biomèdica de Girona (IDIBGI), 17190 Girona, Spain; 4 Primary Care Services, Girona, Catalan Institute of Health (ICS), 17001 Girona, Spain

**Keywords:** peripheral artery disease, diabetes, cardiovascular disease, ankle–brachial index, electronic health records

## Abstract

Cardiovascular prevention is of particular interest in persons with asymptomatic peripheral arterial disease. We aimed to quantify its association with mortality and cardiovascular outcomes, compared to other indicators of high risk. We performed a retrospective cohort study using the Database of the Catalan primary care system (SIDIAP^Q^), for 2006–2015, including 35–85-year-old patients with an ankle–brachial index (ABI) measurement, classified according to the presence of diabetes, cardiovascular disease, and low ABI (<0.9). We calculated the incidences and hazard ratios (HRs) for all-cause mortality, acute myocardial infarction, and ischemic stroke. During a median follow-up of 5.9 years, we analyzed 58,118 persons. The mean (SD) age was 66.6 (10.7) years and 53.4% were men. Compared to the reference group with no diabetes, no previous cardiovascular disease, and normal ankle–brachial index, the HR for all-cause mortality was 1.42 (1.25–1.63) in the group with low ABI, 1.35 (1.26–1.45) in those with diabetes, 1.50 (1.34–1.69) in those with previous cardiovascular disease, and 1.84 (1.68–2.01) in those with low ABI and diabetes. In conclusion, participants with low ABI showed increased mortality, acute myocardial infarction, and ischemic stroke incidence in all the subgroups. Patients with low ankle–brachial index plus diabetes presented increased mortality, acute myocardial infarction, and ischemic stroke risk, all at rates similar to those with previous cardiovascular disease.

## 1. Introduction

Cardiovascular diseases (CVDs) remain a major cause of mortality despite improvements in their prevention and management [1,2]. Prevention of these diseases, a key factor in reducing their mortality and morbidity burden, is grounded in appropriate individual risk assessment [3]. In primary prevention candidates, cardiovascular risk is estimated using risk functions that integrate multiple interacting risk factors [4,5,6]. Some persons, including those with diabetes or previous CVD, are considered to be at high or very high cardiovascular risk without the need of a risk score, and require immediate attention to risk factors [7]. Yet another group, those with asymptomatic disease, is less defined: they have atherosclerosis, including peripheral arterial disease (PAD), but its expression is silent, although harmful, all the same [8]. This is a group of high interest because early detection of asymptomatic disease would allow immediate implementation of preventative measures [9]. 

Asymptomatic PAD can be detected with the ankle–brachial index (ABI), a first screening method after clinical examination [9]. The ABI is the ratio of the systolic blood pressure measured at the ankle to that measured at the brachial artery [8]. It is a simple office-based test with high availability, reproducibility, and cost-effectiveness [8], although it is not exempt from certain limitations [8], and it is readily available in primary care practices, which could be the optimal setting for the screening of this disease. 

The presence of low ABI, defined as <0.9, has been proposed as a modifier of total cardiovascular risk [7]. This association has been assessed in general population, independently from the Framingham risk score [10]; in persons with diabetes [11,12]; and in persons with previous history of coronary artery disease, in addition to diabetes mellitus and traditional risk factors [13]. To date, no studies have examined the extent to which the impact of low ABI is affected by the simultaneous presence or absence of conditions known to increase risk, like diabetes and previous CVD.

Accordingly, we sought to compare the association of low ABI with mortality, acute myocardial infarction (AMI), and ischemic stroke (IS) in a variety of population subgroups according to the baseline presence or absence of diabetes and previous CVD. 

## 2. Methods

### 2.1. Data Source

Data were obtained from the System for the Development of Research in Primary Care (SIDIAP^Q^). This is a database that contains longitudinal information on demographic data, clinical diagnoses coded according to the International Classification of Diseases—10th revision (ICD-10), referral and hospital discharge information coded using ICD-9, laboratory tests, and treatments (drug prescriptions and drug invoicing at any community pharmacy), at an individual and ecological level. Data are standardized, quality-controlled, anonymized, and structured for research purposes. Identifiers are encoded to ensure confidentiality of personal data of patients in the 274 Primary Care Practices (a total of 1365 general practitioners) managed by the Catalan Institute of Health throughout Catalonia. Completeness and continuity are externally assessed. Only records that meet pre-defined data quality standards are included in SIDIAP^Q^, which compiles information on nearly 2 million patients, yielding around 20 million person-years for the period 2005–2015. These high-quality data are representative of the geographical, age, and sex distributions of the population of Catalonia [14], particularly for cardiovascular risk factors and CVD [15], and have been widely used in epidemiological research [16,17,18,19]. Ethics approval to use SIDIAP^Q^ data for observational research was obtained from the Ethics Committee for Clinical Research IDIAP Jordi Gol (P14/052).

### 2.2. Study Design and Participants

This retrospective cohort study included records of patients aged 35 to 85 years with an ABI measurement recorded in the SIDIAP^Q^ database during the recruitment period between January 2006 and December 2011; the date of first ABI measurement defined study entry date. We included patients with normal ABI at entry, defined as 0.9 ≤ ABI < 1.3, or with low ABI, defined as ABI < 0.9. We excluded data from persons with high ABI, defined as ABI ≥ 1.3. ABI values can be high due to medial arterial calcification, especially in persons with diabetes, and this can concur with certain degree of atherosclerosis; thus, we considered this group should be studied separately concerning the purpose of this study, to avoid confusion [20]. To prevent inclusion of persons with symptomatic PAD, we also excluded persons with low ABI who additionally had (i) a prescription of any drug related to intermittent claudication (cilostazol, pentoxifylline, buflomedil, or naftidrofuryl); (ii) any symptom of intermittent claudication detected by thorough review of uncoded information in the attending physician’s notes; or (iii) an ABI < 0.4, because a patient with such level of ABI would very likely be symptomatic. Finally, type I diabetes was an exclusion criterion.

Follow-up extended until an outcome occurred, and censoring applied to participants whose data were transferred out of the SIDIAP^Q^ reference area or at the end of the study period, 31 December 2015. This guaranteed a minimum of 4 years of data for each participant.

### 2.3. Exposure and Outcomes

ABI records followed the official Primary Care Services protocol to standardize ABI measurements [9]. This protocol states that the systolic blood pressure has to be measured in each arm and each ankle just above the malleoli with Doppler probes. The higher value of the dorsalis pedis and the tibial posterior arteries of each leg is divided by the higher value of the systolic blood pressure of the arm. The resulting lower value is the ABI [8].

Participants who fulfilled inclusion criteria were classified into eight groups according to exposure at baseline, defined by ABI category and previous history of CVD and/or diabetes: (1) diabetes, prior CVD, and low ABI values; (2) diabetes and prior CVD, but normal ABI; (3) diabetes and low ABI; (4) prior CVD and low ABI; (5) prior CVD alone; (6) low ABI alone; (7) diabetes alone; (8) no diabetes, no prior CVD, and normal ABI (the reference group). Previous history of CVD included AMI, angina, stroke, and transient ischemic attack. Diabetes at baseline was defined with diagnosis of diabetes or treatment with drugs used in diabetes (coded as a10 in the Anatomical Therapeutic Chemical (ATC) Classification System). The outcomes assessed were all-cause mortality, AMI, and IS.

### 2.4. Covariates

We characterized the population and included potential modifiers of the low ABI association with all-cause mortality, AMI, and IS [5,21,22,23,24,25,26], describing age, sex, smoking habit, continuous variables: body mass index (BMI) -calculated as weight divided by squared height-, systolic and diastolic blood pressure (BP), pulse pressure, low (LDL) and high (HDL) density lipoprotein cholesterol, triglycerides; we considered the last record up to one year previous to entry date (72% to 85% of the population had a record up to 6 months previous to entry date). We also described the following comorbidities: hypertension, atrial fibrillation, malignant neoplasms, chronic kidney disease, and chronic obstructive pulmonary disease. We considered the presence of a condition in a patient if such was recorded previously to entry date. Finally, we accounted for the following medications: diuretics, beta blocking agents, calcium channel blockers, agents acting on the renin–angiotensin system, statins, other lipid modifying agents, and aspirin. A person was defined as treated if they had a purchase record of a given medication up to 6 months previous to the entry date (87% to 93% of the population had a purchase record up to 3 months previous to entry date).

### 2.5. Statistical Analysis

Continuous variables were expressed as mean and standard deviation (SD), or median (1st and 3rd quartiles); and categorical variables as percentages.

To avoid the potential selection bias that may occur when excluding participants with missing values [27], we performed multiple imputation by chained equations of the variables with missing values; we generated 50 imputation tables with 50 iterations each [28]. The population characteristics conceded plausibility to the missing-at-random assumption, and we also performed sensitivity analysis with the complete-case population [29] for comparison.

Raw incidence rates per 1000 person-years of all-cause mortality, AMI, and IS were calculated by exposure group. Cox proportional hazard models were used to analyze the adjusted association of low ABI values with all-cause mortality, AMI, and IS. For all outcomes, a set of candidate variables for adjustment was considered, based on the literature. Initially, we assessed the unadjusted association of the categories of exposure with each outcome. We then tested the association by including the categories of exposure and each candidate variable in the model. Both associations were compared using the standardized difference. We sequentially increased the number of adjusting variables by including the variable with the highest standardized difference. This greedy process was repeated until the standardized difference for the association when adding a candidate variable was lower than 0.10. All analyses were carried out using R-software [30] (version 3.5.1; R Foundation for Statistical Computing, Vienna, Austria), including MICE v2.15 package for multiple imputation [31].

## 3. Results

**Figure 1:**
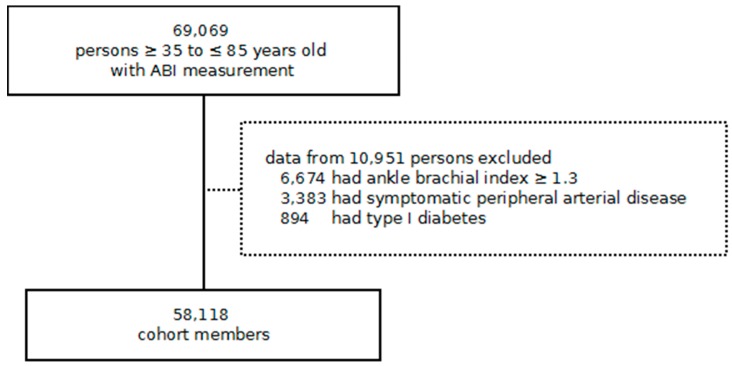
Study flowchart. ABI indicates ankle–brachial index.

During the recruitment period, SIDIAP^Q^ contained data of 69,069 individuals aged 35 to 85 years who had an ABI measurement, 58,118 of which fulfilled inclusion criteria (Figure 1); 41,297 (71.1%) had diabetes, 11,812 (20.3%) had previous CVD, and 10,684 (18.4%) had low ABI. Median (1st quartile, 3rd quartile) follow-up for the whole study population was 5.9 (4.7, 7.6) years. Follow-up was lost for 914 individuals due to transfer from the SIDIAP^Q^ reference area.

We imputed the missing values of weight, height, systolic BP, pulse pressure, glucose, total cholesterol, HDL cholesterol, LDL cholesterol, and triglycerides. Appendix A Table A1 quantifies the missing variables and displays a comparison of the complete cases and imputed datasets for the whole study population. The maximum percentage of missing values was 32.8% for BMI. Although the participants’ characteristics in both subsets were very similar, the subset of complete cases tended to be in slightly worse condition, i.e., higher percentage of patients with hypertension and being treated with antidiabetic drugs, agents acting on the renin–angiotensin system, statins, or with aspirin.

Mean age (SD) of the whole study population was 66.6 (10.7) years old, and 31,064 (53.4%) were men. The groups with low ABI included a higher percentage of smokers, greater prevalence of chronic kidney disease and chronic obstructive pulmonary disease, and a slightly higher mean pulse pressure compared to the groups with normal ABI (Table 1). The groups with low ABI and no previous CVD had a higher percentage of persons taking aspirin and a greater prevalence of atrial fibrillation compared to the groups with normal ABI and no previous CVD. The groups with previous CVD had higher mean values of pulse pressure and a higher percentage of men, persons with hypertension, smokers, and patients receiving treatment with the considered medications, compared to the groups with no previous CVD. Persons in the groups with no previous CVD who had diabetes showed lower total and LDL cholesterol mean values. Finally, the groups with diabetes included a higher percentage of persons with hypertension, with chronic kidney disease, and being treated with the medications considered compared to the groups with no diabetes; this was observed especially in participants with additional previous CVD (Table 1).

Overall, 8382 participants died during the follow-up period, an all-cause mortality incidence of 23.8 (95% CI 23.3, 24.3) per 1000 person-years; 2154 had an AMI, an incidence rate of 6.2 (6.0, 6.5) per 1000 person-years; and 3922 had IS, an incidence rate of 11.5 (11.1, 11.8) per 1000 person-years. The incidences of the considered outcomes by categories of exposure were highest in the group with diabetes, prior CVD, and low ABI values, and lowest in the reference group (no diabetes, no prior CVD, and normal ABI values) (Table 2).

**Figure 2:**
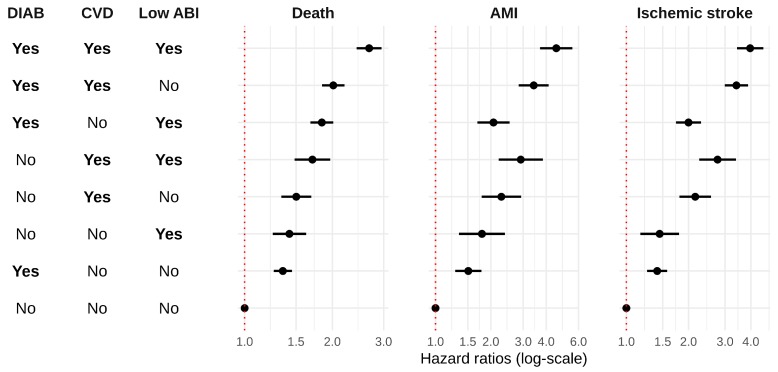
Hazard ratios and 95% confidence intervals for all-cause mortality, acute myocardial infarction, and ischemic stroke by groups of exposure. ABI indicates ankle–brachial index; AMI, acute myocardial infarction; CVD previous cardiovascular disease; DIAB, diabetes.

The risk of all-cause mortality, AMI, and IS was compared between categories of exposure using the group with no diabetes, no previous CVD, and normal ABI as reference. Figure 2 shows the exposure groups by increasing adjusted HR for all-cause mortality, AMI, and IS. The risk for all the considered outcomes was highest in the group with diabetes, previous CVD, and low ABI. Low ABI alone showed an increase in all-cause mortality risk compared to the reference group, with a hazard ratio (HR) of 1.42 (1.25, 1.63), similar to the group with diabetes alone, HR 1.35 (1.26, 1.45), and slightly lower than in the group with previous CVD alone, HR 1.50 (1.34, 1.69). This latter group had lower all-cause mortality risk than participants with low ABI and diabetes, who had a HR of 1.84 (1.68, 2.01), and both confidence intervals nearly overlapped; the group with previous CVD alone and the group with low ABI and diabetes had similar values for AMI and IS. The trends for mortality in the rest of the groups were similar to AMI and IS. Table A2 and Table A3 detail the unadjusted and adjusted HRs (95% CI), respectively, for all the categories and outcomes. Appendix A Table A4 shows the HRs of the adjustment variables for each outcome.

Sensitivity analyses restricted to the complete-cases dataset gave similar results (Table A5, Table A6 and Table A7). Appendix A Table A5 presents data from the complete-cases dataset by population groups. As in the overall population, the subset of complete cases tended to be in slightly worse condition, but the group distribution was similar to the imputed dataset. Appendix A Table A6 presents the raw incidences in the subset of complete cases, which had a similar pattern to the imputed dataset; given the difference in sample sizes, the confidence intervals were slightly wider in the complete-cases subset than in the imputed dataset. Appendix A Table A7 shows the adjusted HRs for the complete- cases subset of population. The HRs were slightly higher for all-cause mortality and slightly lower for AMI and IS in the subset of complete cases compared to the imputed data. We also performed sensitivity analyses considering persons under and over 75 years of age, and forcing chronic kidney disease in the models—since it is a strong risk factor for AMI and stroke—which presented comparable results. 

## 4. Discussion

We found that the presence of low ABI in asymptomatic persons was associated with increased all-cause mortality, AMI, and IS risk in all the studied subgroups, aside from the presence of diabetes or previous CVD. This increase was observed even in participants at highest risk (with both diabetes and previous CVD). At the other end of the risk array, persons with ABI alone presented an incidence of all the considered outcomes similar to persons with diabetes alone; this incidence was lower than in persons with previous CVD alone. Of note, participants with low ABI plus diabetes showed an increase in mortality, AMI, and IS risk similar to those with previous CVD.

Previous studies have evidenced the relation of low ABI with mortality or cardiovascular outcomes worldwide. In Central Africa, HR estimates for low ABI (≤0.9) were 1.86 for mortality in a population aged ≥65 years, 10% of whom had diabetes and 7% whom had a history of myocardial infarction [32]. In Japan, HR estimates for abnormal ABI (<0.9 or >1.4) were 2.01 for major adverse cardiovascular events in a population with end-stage kidney disease, 38% of whom had diabetes and 28% whom had prior coronary revascularization [33]. In France, a HR for total mortality of 1.46 was reported in a population prior to coronary artery bypass grafting [34]. Comparison between studies is difficult, not only because of the differences in the design, but also in the characteristics of the populations.

The increase in all-cause mortality, AMI, and IS risk conferred by low ABI in asymptomatic participants with previous CVD and/or diabetes is compatible with low ABI being a surrogate of vascular damage in the context of polyvascular disease; indeed, low ABI has been reported to be useful in identifying this condition [35,36]. In addition to corroborating these findings, our results quantify the risk increase contributed by polyvascular damage, and are in line with previous recommendations to use ABI for the detection of asymptomatic PAD in persons with previous CVD and diabetes, both in isolation or when coexistent [37].

The damage associated with low ABI would be of interest not only in the context of polyvascular disease but also in persons with low ABI alone, in whom we found an all-cause mortality risk as high as in persons with diabetes. To date, evidence to recommend screening with ABI in asymptomatic persons has been deemed insufficient [38], even though some studies evidenced the benefit of screening of asymptomatic population at high risk which, although modest, should be taken into consideration [39,40]. Our results would support the need for this screening, although further studies specifically designed for this purpose would be of high interest. Even more, risk functions have been developed to help optimize the selection of candidates for screening of asymptomatic individuals [41]. Given the serious consequences of PAD, its early diagnosis in asymptomatic stages is crucial in terms of cardiovascular risk and to prevent its progression in the form of foot ulceration, gangrene, and eventual amputation of the affected part of the extremity [42].

The comparison of the group with previous CVD and the group with diabetes and additional low ABI merits special discussion. In several reports [43,44,45,46], patients with diabetes were considered as comparable to those with previous CVD, although others have argued against it [47,48]. This controversy was resolved with a tendency to ascribe higher risk to patients with previous CVD; consequently, recommendations and treatments for cardiovascular prevention were not as aggressive in patients with diabetes and no previous CVD [47]. However, some studies tried to reconcile these two positions on risk among patients with diabetes. Previous CVD conferred a risk similar to having diabetes in patients who required glucose-lowering medication [43], used insulin or had albuminuria [49], or if they had a longer duration of diabetes, i.e., 10–15 years [50,51]. Our study adds low ABI values to these findings. Further studies that examined the impact of variations of ABI over time would be of interest as to provide additional prognosis information.

The present analysis was performed with a database originated from clinical records, a ‘real world’ assessment [9] of all-cause mortality, AMI, and IS, based on a high number of participants; this provided sufficient power to stratify the population into groups of exposure that could be directly analyzed and compared. Working with SIDIAP^Q^ also provided a high absolute number of outcomes and a long follow-up period. The study population should be taken into consideration when extrapolating our results. It was a population with risk factors for atherosclerotic disease (the mean age was 66.6 years, where 67.9% of participants had hypertension, 25.8% were smokers or had a history of smoking, 71.1% had diabetes, and 20.3% had previous CVD), to whom an ABI measurement would be recommended according to the prevention guidelines [4,52,53,54,55].

Our findings should be interpreted in light of some limitations. First, we did not describe and analyze hemoglobin A1c levels or the time since previous CVD because the study design required the use of the same variables for all patients in order to directly compare the groups and the risk of the outcomes. Some groups included participants with no diabetes or previous CVD and, thus, the amount of missing values for hemoglobin A1c would have been unacceptable; in addition, some participants did not have data regarding time since previous CVD. Second, potential residual confounding can never be totally dismissed, even though we adjusted for important cardiovascular risk factors. Third, poor quality of the data could generate misclassification in studies with electronic medical records, but the presence of cardiovascular risk factors and outcomes has been previously validated in SIDIAP [15]. Fourth, the presence of missing data can influence the results but, in this study, the maximum percentage of missing values for a variable was 32.8%, and the characteristics of the complete-case analyses did not differ from imputed data. Finally, SIDIAP^Q^ does not contain records on cause of death and, thus, we could not assess cardiovascular death specifically, but our results indicate that it could be partially explained, in most of the studied subgroups, by the cardiovascular outcomes considered.

In conclusion, low ABI was associated with increased all-cause mortality, AMI, and IS risk at rates similar to diabetes but less than previous CVD. The risk of AMI or IS in persons with diabetes was similar to that in persons with previous CVD, provided low ABI was also present. These findings will contribute to improve awareness on the risk associated with low ABI in asymptomatic patients, and support the need for further studies to elucidate who should be screened in order to optimize CVD prevention.

## Figures and Tables

**Table 1:** Baseline characteristics of the study population according to diabetes, prior CVD, and ankle–brachial index.

	No Diabetes				Diabetes			
	No CVD		Prior CVD		No CVD		Prior CVD	
	No LOW.ABI	LOW.ABI ^a^	No LOW.ABI	LOW.ABI	No LOW.ABI	LOW.ABI	No LOW.ABI	LOW.ABI
*n* (%)	11,850 (20.4%)	1880 (3.2%)	2064 (3.6%)	1027 (1.8%)	27,434 (47.2%)	5142 (8.8%)	6086 (10.5%)	2635 (4.5%)
Age, years	64.6 (11.4)	66.3 (11.9)	69.7 (9.8)	69.8 (10.0)	65.8 (10.5)	67.9 (10.4)	69.9 (9.1)	70.7 (9.1)
Male, *n* (%)	5353 (45.2%)	1068 (56.8%)	1350 (65.4%)	799 (77.8%)	13,853 (50.5%)	2819 (54.8%)	3978 (65.4%)	1844 (70.0%)
Smoker, *n* (%)	2786 (23.5%)	711 (37.8%)	648 (31.4%)	525 (51.1%)	6172 (22.5%)	1458 (28.4%)	1762 (29.0%)	948 (36.0%)
Weight, kg	76.3 (14.4)	77.2 (15.4)	77.1 (13.1)	76.0 (13.4)	79.0 (14.8)	78.9 (14.8)	79.1 (13.9)	78.1 (13.8)
Height, cm	161.1 (9.3)	161.8 (9.3)	162.3 (8.7)	162.7 (8.3)	161.2 (9.5)	161.2 (9.4)	162.2 (9.0)	162.2 (8.9)
Body mass index, kg/m^2^	29.4 (4.7)	29.4 (5.0)	29.3 (4.3)	28.7 (4.2)	30.4 (5.0)	30.4 (5.1)	30.1 (4.5)	29.7 (4.5)
Systolic BP, mmHg	133.1 (15.3)	135.1 (16.2)	132.6 (15.7)	136.2 (18.3)	135.8 (15.4)	138.5 (17.2)	135.2 (16.3)	137.7 (17.5)
Diastolic BP, mmHg	77.9 (9.4)	77.5 (10.2)	75.6 (9.8)	75.1 (10.8)	77.3 (9.4)	76.4 (9.9)	74.3 (9.6)	73.5 (10.4)
Pulse pressure, mmHg	55.2 (13.3)	57.6 (14.2)	57.0 (14.1)	61.1 (15.8)	58.4 (14.1)	62.0 (15.9)	60.9 (14.8)	64.2 (16.0)
Total cholesterol, mmol/L	5.5 (0.9)	5.5 (1.0)	5.0 (1.0)	5.2 (1.1)	5.1 (1.0)	5.1 (1.0)	4.6 (1.0)	4.7 (1.0)
LDL cholesterol, mmol/L	3.4 (0.8)	3.4 (0.9)	3.0 (0.9)	3.2 (0.9)	3.0 (0.8)	3.0 (0.8)	2.6 (0.8)	2.7 (0.8)
HDL cholesterol, mmol/L	1.5 (0.5)	1.4 (0.5)	1.4 (0.4)	1.4 (0.5)	1.4 (0.4)	1.3 (0.4)	1.3 (0.4)	1.2 (0.4)
Triglycerides, mmol/L	1.4 (0.9)	1.5 (0.9)	1.5 (0.9)	1.6 (0.9)	1.7 (1.1)	1.8 (1.2)	1.7 (1.0)	1.8 (1.3)
Glucose, mmol/L	5.4 (0.9)	5.4 (0.9)	5.4 (0.8)	5.5 (0.9)	8.3 (2.8)	8.6 (3.0)	8.1 (2.7)	8.4 (2.9)
Comorbidities, *n* (%)								
Hypertension	6963 (58.8%)	1134 (60.3%)	1476 (71.5%)	743 (72.3%)	18,350 (66.9%)	3740 (72.7%)	4874 (80.1%)	2178 (82.7%)
Atrial fibrillation	448 (3.8%)	109 (5.8%)	275 (13.3%)	135 (13.1%)	1218 (4.4%)	342 (6.7%)	772 (12.7%)	366 (13.9%)
Malignant neoplasm	850 (7.2%)	163 (8.7%)	208 (10.1%)	115 (11.2%)	2173 (7.9%)	425 (8.3%)	618 (10.2%)	293 (11.1%)
Chronic kidney disease	325 (2.7%)	77 (4.1%)	152 (7.4%)	96 (9.3%)	1069 (3.9%)	276 (5.4%)	600 (9.9%)	350 (13.3%)
COPD	991 (8.4%)	288 (15.3%)	377 (18.3%)	268 (26.1%)	2328 (8.5%)	661 (12.9%)	978 (16.1%)	551 (20.9%)
Medication, *n* (%)								
Antidiabetic therapy	0 (0.0%)	0 (0.0%)	0 (0.0%)	0 (0.0%)	21,388 (78.0%)	4193 (81.5%)	5014 (82.4%)	2251 (85.4%)
Diuretics	2565 (21.6%)	483 (25.7%)	606 (29.4%)	303 (29.5%)	6369 (23.2%)	1414 (27.5%)	2102 (34.5%)	998 (37.9%)
Beta-blockers	1234 (10.4%)	232 (12.3%)	651 (31.5%)	280 (27.3%)	3382 (12.3%)	701 (13.6%)	2557 (42.0%)	977 (37.1%)
Calcium channel blockers	1258 (10.6%)	243 (12.9%)	506 (24.5%)	253 (24.6%)	4516 (16.5%)	1009 (19.6%)	2024 (33.3%)	912 (34.6%)
Agents acting on the renin angiotensin system	4719 (39.8%)	844 (44.9%)	1144 (55.4%)	562 (54.7%)	15,536 (56.6%)	3281 (63.8%)	4311 (70.8%)	1941 (73.7%)
Other antihypertensives	321 (2.7%)	62 (3.3%)	87 (4.2%)	46 (4.5%)	1257 (4.6%)	291 (5.7%)	450 (7.4%)	207 (7.9%)
Statins	2726 (23.0%)	503 (26.8%)	1154 (55.9%)	538 (52.4%)	12,207 (44.5%)	2445 (47.5%)	4339 (71.3%)	1837 (69.7%)
Other lipid lowering agents	345 (2.9%)	72 (3.8%)	118 (5.7%)	63 (6.1%)	1875 (6.8%)	341 (6.6%)	686 (11.3%)	299 (11.3%)
Aspirin	1013 (8.5%)	326 (17.3%)	1137 (55.1%)	552 (53.7%)	7366 (26.8%)	1754 (34.1%)	4012 (65.9%)	1692 (64.2%)
Follow-up, years, (1st quartile, 3rd quartile)	6.2 (4.8, 7.9)	5.7 (4.6, 7.4)	5.8 (4.6, 7.5)	5.6 (4.5, 7.2)	6.1 (4.8, 7.5)	5.8 (4.7, 7.5)	5.6 (4.6, 7.2)	5.3 (4.2, 7.1)
Lost to follow-up, *n* (%) ^b^	194 (1.6%)	30 (1.6%)	33 (1.6%)	16 (1.6%)	419 (1.5%)	92 (1.8%)	89 (1.5%)	41 (1.6%)

^a^ ABI < 0.9. ^b^ With respect to the number of participants in that group. Values are mean (standard deviation), unless otherwise specified. ABI indicates ankle–brachial index; BP, blood pressure; CVD, cardiovascular disease; COPD, chronic obstructive pulmonary disease; HDL, high density lipoprotein; LDL, low density lipoprotein; *n*, number of persons.

**Table 2:** Raw incidences of all-cause mortality, acute myocardial infarction, and ischemic stroke by population groups.

Group			All-Cause Mortality		Acute Myocardial Infarction		Ischemic Stroke	
Diabetes	CVD	Low ABI ^a^	Events ^b^	Incidence Rate ^c^ (95% CI)	Events	Incidence Rate (95% CI)	Events	Incidence Rate (95% CI)
**Yes**	**Yes**	**Yes**	873	60.8 (56.9, 65.0)	290	21.4 (19.0, 24.0)	431	32.7 (29.8, 36.0)
**Yes**	**Yes**	No	1401	40.2 (38.1, 42.4)	495	14.8 (13.6, 16.2)	834	25.6 (23.9, 27.4)
**Yes**	No	**Yes**	965	31.3 (29.4, 33.4)	208	6.9 (6.0, 7.9)	410	13.8 (12.5, 15.2)
No	**Yes**	**Yes**	258	44.2 (39.1, 49.9)	75	13.3 (10.6, 16.7)	127	23.2 (19.5, 27.7)
No	**Yes**	No	400	32.7 (29.7, 36.1)	105	8.8 (7.3, 10.7)	190	16.3 (14.1, 18.8)
No	No	**Yes**	281	25.4 (22.6, 28.5)	62	5.7 (4.4, 7.3)	104	9.7 (8.0, 11.7)
**Yes**	No	No	3146	18.7 (18.0, 19.3)	728	4.4 (4.1, 4.7)	1402	8.5 (8.1, 9.0)
No	No	No	1058	14.1 (13.3, 15.0)	191	2.6 (2.2, 3.0)	424	5.7 (5.2, 6.3)

^a^ ABI < 0.9. ^b^ Number of events. ^c^ Expressed per 1000 person-years. ABI indicates ankle–brachial index; CI, confidence interval; CVD, cardiovascular disease.

## Appendix A

### Appendix A.1. Methods

Validation of the Imputation Process [27,29]

In the multiple imputation stage, we checked for normality, correlations, and collinearity between variables with missing data and variables that could be included in the multiple imputation. Next, we identified variables related to missing values and/or to missing variables.

The imputation models included the following variables: age, sex, ankle–brachial index (ABI), weight, height, natural logarithm (ln) of systolic blood pressure, ln (pulse pressure), ln (glucose), ln (total cholesterol), ln (high density lipoprotein cholesterol), ln (low density lipoprotein cholesterol), ln (triglycerides), smoking status, hypertension, hypercholesterolemia, arthritis, asthma, chronic obstructive pulmonary disease, atrial fibrillation, malignant neoplasms, chronic kidney disease, hypothyroidism, previous history of acute myocardial infarction (AMI), angina pectoris, transient ischemic attack, stroke, and medications (antidiabetic drugs, diuretics, beta blocking agents, calcium-channel blockers, agents acting on the renin–angiotensin system, other antihypertensive agents, corticosteroids for systemic use, psycholeptics, psychoanaleptics, statins, other lipid-lowering drugs, and aspirin. We also included the censoring indicator and the Nelson–Aalen estimate of the cumulative hazard function for the time to cardiovascular disease [28].

The ln transformation was applied to some variables, as indicated in the list above, to improve the normality of the distribution and to avoid the unlikely possibility of imputing any negative numbers. After imputation, variables were converted back to their original scale.

Appendix ATable A1 shows the number (percentage) of missing values for each variable of interest and the baseline characteristics of the whole imputed population, and of the subset with complete cases.

**Table A1:** Missing counts and comparison of baseline characteristics of the whole study population for the imputed and complete-cases datasets.

Variable	Missing Counts ^a^	Imputed *n* = 58,118	Complete Cases *n* = 28,206
Age, years	-	66.6 (10.7)	67.3 (10.1)
Male, *n* (%)	-	31,064 (53.4%)	15,012 (53.2%)
Smoker, *n* (%)	-	15,010 (25.8%)	7086 (25.1%)
Weight, kg	14,604 (25.1%)	78.3 (14.6)	78.2 (13.9)
Height, cm	18,921 (32.6%)	161.4 (9.3)	161.1 (9.2)
Body mass index, kg/m^2^	19,073 (32.8%)	30.0 (4.8)	30.1 (4.7)
Systolic BP, mmHg	7652 (13.2%)	135.4 (15.9)	135.7 (15.5)
Diastolic BP, mmHg	7652 (13.2%)	76.8 (9.7)	76.5 (9.5)
Pulse pressure, mmHg	7652 (13.2%)	58.6 (14.5)	59.2 (14.3)
Total cholesterol, mmol/L	14,366 (24.7%)	5.1 (1.0)	5.1 (1.0)
LDL cholesterol, mmol/L	18,625 (32.0%)	3.0 (0.9)	3.0 (0.8)
HDL cholesterol, mmol/L	18,179 (31.3%)	1.4 (0.4)	1.3 (0.4)
Triglycerides, mmol/L	16,954 (29.2%)	1.7 (1.1)	1.7 (1.0)
Glucose, mmol/L	13,746 (23.7%)	7.5 (2.8)	7.7 (2.7)
Comorbidities, *n* (%)			
Hypertension	-	39,458 (67.9%)	20,624 (73.1%)
Atrial fibrillation	-	3665 (6.3%)	1818 (6.4%)
Malignant neoplasm	-	4845 (8.3%)	2292 (8.1%)
Chronic kidney disease	-	2945 (5.1%)	1502 (5.3%)
COPD	-	6442 (11.1%)	3169 (11.2%)
Acute myocardial infarction		6922 (11.91%)	3277 (11.62%)
Angina		2680 (4.61%)	1395 (4.95%)
Stroke		2913 (5.01%)	1475 (5.23%)
Transient ischemic attack		1640 (2.82%)	831 (2.95%)
Medication, *n* (%)			
Antidiabetic therapy	-	32,846 (56.5%)	18,480 (65.5%)
Diuretics	-	14,840 (25.5%)	7627 (27.0%)
Beta-blockers	-	10,014 (17.2%)	5191 (18.4%)
Calcium channel blockers	-	10,721 (18.4%)	5787 (20.5%)
Agents acting on the renin–angiotensin system	-	32,338 (55.6%)	17,418 (61.8%)
Other antihypertensives	-	2721 (4.7%)	1378 (4.9%)
Statins	-	25,749 (44.3%)	14,419 (51.1%)
Other lipid-lowering agents	-	3799 (6.5%)	1995 (7.1%)
Aspirin	-	17,852 (30.7%)	9636 (34.2%)
Follow-up, years (1st quartile, 3rd quartile)	-	5.9 (4.7, 7.6)	5.8 (4.7, 7.2)
Lost to follow-up, *n* (%)	-	914 (1.6%)	421 (1.5%)

Abbreviations: BP, blood pressure; COPD, chronic obstructive pulmonary disease; HDL, high density lipoprotein; LDL, low density lipoprotein; *n*, number of persons; SD, standard deviation. Values are mean (standard deviation), unless otherwise specified. ^a^ *n* (%).

**Table A2:** Unadjusted hazard ratios (95% confidence intervals) for all-cause mortality, acute myocardial infarction, and ischemic stroke, by groups.

Group			Hazard Ratio (95% Confidence Interval)		
Diabetes	CVD	Low ABI	All-Cause Mortality	AMI	Ischemic Stroke
**Yes**	**Yes**	**Yes**	2.74 (2.32, 3.24)	4.51 (3.26, 6.23)	3.89 (3.09, 4.89)
**Yes**	**Yes**	No	2.08 (1.78, 2.42)	3.28 (2.41, 4.46)	3.38 (2.74, 4.17)
**Yes**	No	**Yes**	2.11 (1.81, 2.47)	1.83 (1.31, 2.54)	1.87 (1.49, 2.34)
No	**Yes**	**Yes**	1.58 (1.21, 2.07)	1.96 (1.14, 3.36)	2.44 (1.69, 3.53)
No	**Yes**	No	1.66 (1.34, 2.07)	1.98 (1.27, 3.10)	1.85 (1.35, 2.54)
No	No	**Yes**	1.54 (1.21, 1.97)	1.76 (1.08, 2.88)	1.32 (0.90, 1.92)
**Yes**	No	No	1.52 (1.33, 1.74)	1.47 (1.11, 1.94)	1.44 (1.19, 1.74)
No	No	No	Reference	Reference	Reference

Abbreviations: ABI, ankle–brachial index; AMI, acute myocardial infarction; CVD, cardiovascular disease.

**Table A3:** Adjusted hazard ratios (95% confidence intervals) for all-cause mortality, acute myocardial infarction, and ischemic stroke, by groups.

Group			Hazard Ratio (95% Confidence Interval)		
Diabetes	CVD	Low ABI	All-Cause Mortality	AMI	Ischemic Stroke
**Yes**	**Yes**	**Yes**	2.68 (2.42, 2.95)	4.54 (3.71, 5.56)	3.97 (3.43, 4.59)
**Yes**	**Yes**	No	2.01 (1.84, 2.20)	3.42 (2.84, 4.13)	3.41 (2.99, 3.88)
**Yes**	No	**Yes**	1.84 (1.68, 2.01)	2.07 (1.69, 2.53)	2.00 (1.74, 2.30)
No	**Yes**	**Yes**	1.71 (1.49, 1.97)	2.91 (2.21, 3.84)	2.76 (2.25, 3.39)
No	**Yes**	No	1.50 (1.34, 1.69)	2.28 (1.78, 2.92)	2.15 (1.81, 2.57)
No	No	**Yes**	1.42 (1.25, 1.63)	1.79 (1.34, 2.39)	1.45 (1.17, 1.80)
**Yes**	No	No	1.35 (1.26, 1.45)	1.51 (1.28, 1.78)	1.41 (1.26, 1.58)
No	No	No	Reference	Reference	Reference

Abbreviations: ABI, ankle–brachial index; AMI, acute myocardial infarction; CVD, cardiovascular disease.

**Table A4:** Variables of adjustment in the Cox proportional hazard models for each outcome.

Variable of Adjustment	Outcome		
	Mortality	AMI	Stroke
Age	√	√	√
Age^2^	√	√	√
Male	√	√	√
Smoker	√	√	√
BMI	√	√	√
Pulse pressure	√	√	√
LDL cholesterol	√	√	
HDL cholesterol	√	√	√
Triglycerides		√	√
Comorbidities			
Hypertension			√
Atrial fibrillation	√	√	√
Malignant neoplasm	√		
Chronic kidney disease	√		
COPD	√	√	√
Medications			
Diuretics	√	√	√
Beta-blockers	√	√	√
Calcium channel blockers	√	√	√
Agents acting on the renin angiotensin system	√	√	√
Statins	√	√	√
Other lipid lowering agents		√	
Aspirin	√	√	√

Abbreviations: AMI, acute myocardial infarction; BMI, body mass index; COPD, chronic obstructive pulmonary disease; HDL, high density lipoprotein; LDL, low density lipoprotein.

**Table A5:** Baseline characteristics of the study population according to diabetes, prior CVD, and ankle–brachial index. Complete cases.

	No Diabetes				Diabetes			
	No CVD		Prior CVD		No CVD		Prior CVD	
	No LOW.ABI	LOW.ABI ^a^	No LOW.ABI	LOW.ABI	No LOW.ABI	LOW.ABI	No LOW.ABI	LOW.ABI
*n* (%)	3704 (13.1%)	645 (2.3%)	736 (2.6%)	337 (1.2%)	15,373 (54.5%)	2696 (9.6%)	3304 (11.7%)	1411 (5.0%)
Age, years	66.8 (10.3)	68.4 (10.5)	70.4 (8.7)	71.1 (9.5)	66.1 (10.2)	68.2 (10.0)	70.0 (8.9)	70.9 (8.8)
Male, *n* (%)	1650 (44.5%)	383 (59.4%)	476 (64.7%)	256 (76.0%)	7621 (49.6%)	1500 (55.6%)	2136 (64.6%)	990 (70.2%)
Smoker, *n* (%)	798 (21.5%)	220 (34.1%)	216 (29.3%)	163 (48.4%)	3417 (22.2%)	780 (28.9%)	971 (29.4%)	521 (36.9%)
Weight, kg	76.3 (13.7)	77.4 (14.7)	76.6 (12.4)	75.2 (11.7)	78.6 (14.1)	78.8 (14.1)	78.8 (13.4)	78.0 (13.6)
Height, cm	160.4 (9.2)	161.5 (9.1)	161.9 (8.5)	162.0 (8.0)	160.9 (9.4)	161.2 (9.2)	162.0 (9.0)	162.0 (8.9)
Body mass index, kg/m^2^	29.6 (4.5)	29.6 (4.8)	29.2 (4.0)	28.6 (3.9)	30.3 (4.8)	30.4 (5.0)	30.0 (4.4)	29.7 (4.5)
Hypertension, *n* (%)	2780 (75.1%)	499 (77.4%)	593 (80.6%)	279 (82.8%)	10,579 (68.8%)	2015 (74.7%)	2694 (81.5%)	1185 (84.0%)
Systolic BP, mmHg	134.4 (14.9)	136.2 (16.3)	133.9 (15.1)	135.8 (17.5)	135.5 (15.1)	138.4 (16.9)	135.2 (15.7)	137.3 (17.2)
Diastolic BP, mmHg	77.7 (9.5)	76.7 (9.9)	75.8 (9.9)	74.0 (10.0)	77.1 (9.3)	76.2 (9.7)	74.1 (9.5)	73.3 (10.2)
Pulse pressure, mmHg	56.7 (13.3)	59.5 (14.7)	58.1 (14.1)	61.8 (15.2)	58.4 (13.8)	62.2 (15.7)	61.0 (14.6)	64.0 (16.1)
Total cholesterol, mmol/L	5.5 (0.9)	5.5 (1.0)	5.0 (1.0)	5.1 (1.0)	5.1 (0.9)	5.1 (1.0)	4.6 (0.9)	4.7 (1.0)
LDL cholesterol, mmol/L	3.4 (0.8)	3.4 (0.9)	2.9 (0.8)	3.1 (0.9)	2.9 (0.8)	3.0 (0.8)	2.6 (0.8)	2.7 (0.8)
HDL cholesterol, mmol/L	1.5 (0.4)	1.4 (0.4)	1.4 (0.4)	1.3 (0.3)	1.3 (0.3)	1.3 (0.3)	1.3 (0.3)	1.2 (0.3)
Triglycerides, mmol/L	1.4 (0.8)	1.5 (0.8)	1.5 (0.8)	1.5 (0.8)	1.7 (1.0)	1.7 (1.0)	1.7 (0.9)	1.7 (1.1)
Glucose, mmol/L	5.4 (0.9)	5.5 (0.9)	5.4 (0.7)	5.4 (0.7)	8.2 (2.7)	8.4 (2.9)	8.0 (2.6)	8.4 (2.9)
Comorbidities, *n* (%)								
Atrial fibrillation	170 (4.6%)	45 (7.0%)	102 (13.9%)	43 (12.8%)	684 (4.4%)	163 (6.0%)	407 (12.3%)	204 (14.5%)
Malignant neoplasm	269 (7.3%)	48 (7.4%)	69 (9.4%)	39 (11.6%)	1180 (7.7%)	206 (7.6%)	330 (10.0%)	151 (10.7%)
Chronic kidney disease	144 (3.9%)	31 (4.8%)	60 (8.2%)	41 (12.2%)	598 (3.9%)	142 (5.3%)	312 (9.4%)	174 (12.3%)
COPD	354 (9.6%)	103 (16.0%)	139 (18.9%)	109 (32.3%)	1295 (8.4%)	338 (12.5%)	532 (16.1%)	299 (21.2%)
Medication, *n* (%)								
Antidiabetic therapy	0 (0.0%)	0 (0.0%)	0 (0.0%)	0 (0.0%)	12,261 (79.8%)	2232 (82.8%)	2755 (83.4%)	1232 (87.3%)
Diuretics	1005 (27.1%)	198 (30.7%)	240 (32.6%)	110 (32.6%)	3664 (23.8%)	745 (27.6%)	1123 (34.0%)	542 (38.4%)
Beta-blockers	473 (12.8%)	91 (14.1%)	239 (32.5%)	107 (31.8%)	1918 (12.5%)	385 (14.3%)	1436 (43.5%)	542 (38.4%)
Calcium channel blockers	536 (14.5%)	108 (16.7%)	207 (28.1%)	109 (32.3%)	2655 (17.3%)	552 (20.5%)	1126 (34.1%)	494 (35.0%)
Agents acting on the renin–angiotensin system	1956 (52.8%)	376 (58.3%)	461 (62.6%)	218 (64.7%)	9090 (59.1%)	1801 (66.8%)	2424 (73.4%)	1092 (77.4%)
Other antihypertensives	125 (3.4%)	35 (5.4%)	35 (4.8%)	18 (5.3%)	675 (4.4%)	161 (6.0%)	229 (6.9%)	100 (7.1%)
Statins	1189 (32.1%)	232 (36.0%)	461 (62.6%)	213 (63.2%)	7448 (48.4%)	1383 (51.3%)	2456 (74.3%)	1037 (73.5%)
Other lipid-lowering agents	137 (3.7%)	30 (4.7%)	41 (5.6%)	27 (8.0%)	1046 (6.8%)	178 (6.6%)	378 (11.4%)	158 (11.2%)
Aspirin	378 (10.2%)	136 (21.1%)	440 (59.8%)	197 (58.5%)	4348 (28.3%)	926 (34.3%)	2272 (68.8%)	939 (66.5%)
Follow-up, years, IQR	5.8 (4.7, 7.5)	5.7 (4.6, 7.2)	5.7 (4.6, 7.2)	5.5 (4.5, 6.9)	5.9 (4.8, 7.3)	5.6 (4.6, 7.1)	5.6 (4.6, 7.1)	5.4 (4.4, 7.0)
Lost to follow-up, *n* (%) ^b^	58 (1.6%)	9 (1.4%)	8 (1.1%)	6 (1.8%)	220 (1.4%)	52 (1.9%)	50 (1.5%)	18 (1.3%)

Abbreviations: ABI, ankle–brachial index; BP, blood pressure; COPD, chronic obstructive pulmonary disease; CVD, cardiovascular disease; HDL, high density lipoprotein; LDL, low density lipoprotein; n, number of persons. Values are mean (standard deviation), unless otherwise specified. ^a^ ABI < 0.9. ^b^ With respect to the number of participants in that group.

**Table A6:** Raw incidences of all-cause mortality, acute myocardial infarction, and ischemic stroke by population groups. Complete cases.

Group			All-Cause Mortality		Acute Myocardial Infarction		Stroke	
Diabetes	CVD	Low ABI ^a^	Events ^b^	Incidence Rate ^c^ (95% CI)	Events	Incidence Rate (95% CI)	Events	Incidence Rate (95% CI)
**Yes**	**Yes**	**Yes**	406	53.0 (48.0, 58.4)	153	21.0 (17.9, 24.6)	229	32.5 (28.5, 36.9)
**Yes**	**Yes**	No	648	34.5 (32.0, 37.3)	248	13.7 (12.1, 15.5)	445	25.3 (23.1, 27.8)
**Yes**	No	**Yes**	428	27.7 (25.2, 30.5)	94	6.2 (5.0, 7.6)	188	12.5 (10.9, 14.5)
No	**Yes**	**Yes**	69	36.4 (28.7, 46.1)	18	9.8 (6.2, 15.6)	38	21.4 (15.5, 29.4)
No	**Yes**	No	124	29.2 (24.5, 34.9)	31	7.4 (5.2, 10.6)	58	14.2 (11.0, 18.4)
No	No	**Yes**	86	23.0 (18.6, 28.4)	22	6.0 (3.9, 9.1)	34	9.3 (6.6, 13.0)
**Yes**	No	No	1510	16.4 (15.6, 17.2)	383	4.2 (3.8, 4.6)	759	8.4 (7.8, 9.0)
No	No	No	260	11.6 (10.3, 13.1)	59	2.7 (2.1, 3.4)	132	6.0 (5.1, 7.1)

^a^ ABI < 0.9. ^b^ Number of events. ^c^ Expressed per 1000 person-years. ABI indicates ankle–brachial index; CI, confidence interval; CVD, cardiovascular disease.

**Table A7:** Adjusted hazard ratios (95% confidence intervals) for all-cause mortality, acute myocardial infarction, and ischemic stroke, by groups. Complete cases.

Group			HR (95% Confidence Interval)		
Diabetes	CVD	Low ABI ^a^	All-Cause Mortality	AMI	Ischemic Stroke
**Yes**	**Yes**	**Yes**	2.74 (2.32, 3.24)	4.51 (3.26, 6.23)	3.89 (3.09, 4.89)
**Yes**	**Yes**	No	2.08 (1.78, 2.42)	3.28 (2.41, 4.46)	3.38 (2.74, 4.17)
**Yes**	No	**Yes**	2.11 (1.81, 2.47)	1.83 (1.31, 2.54)	1.87 (1.49, 2.34)
No	**Yes**	**Yes**	1.58 (1.21, 2.07)	1.96 (1.14, 3.36)	2.44 (1.69, 3.53)
No	**Yes**	No	1.66 (1.34, 2.07)	1.98 (1.27, 3.10)	1.85 (1.35, 2.54)
No	No	**Yes**	1.54 (1.21, 1.97)	1.76 (1.08, 2.88)	1.32 (0.90, 1.92)
**Yes**	No	No	1.52 (1.33, 1.74)	1.47 (1.11, 1.94)	1.44 (1.19, 1.74)
No	No	No	Reference	Reference	Reference

Abbreviations: ABI, ankle–brachial index; AMI, acute myocardial infarction; CVD, cardiovascular disease. ^a^ ABI < 0.9.
